# Inhibition of the complement component C5 and the Toll-like receptor molecule CD14 prevents systemic and local kidney inflammation in mice experiencing brain death

**DOI:** 10.1038/s41598-025-12071-x

**Published:** 2025-07-25

**Authors:** Tom E. Mollnes, Neeltina M. Jager, Petra J. Ottens, Camilla Schjalm, Zwanida J. Veldhuis, Henri G. D. Leuvenink, Søren E. Pischke

**Affiliations:** 1 Department of Immunology, Oslo University Hospital and University of Oslo, Oslo, Norway; 2 Research Laboratory, Nordland Hospital Trust, Bodø, Norway; 3 Department of Anesthesiology, University of Groningen, University Medical Center Groningen, Groningen, The Netherlands; 4 Department of Surgery, University of Groningen, University Medical Center Groningen, Groningen, The Netherlands; 5 Department of Anesthesiology and Intensive Care, Oslo University Hospital, Oslo, Norway

**Keywords:** Brain death, Donor, Inflammation, Complement, CD14, Kidney, Drug discovery, Immunology, Biomarkers, Diseases, Medical research, Nephrology, Pathogenesis

## Abstract

Brain death (BD) induces a systemic inflammation impairing donor organ quality. Complement and Toll-like receptors (TLRs), with the key co-receptor CD14 molecule, are key innate recognition immune systems. We hypothesized that dual inhibition of complement (C5) and TLRs (CD14) will prevent BD-mediated innate immune inflammation. BD was induced in mice either untreated, treated with a C5 inhibitor, a CD14 inhibitor, or both. Blood and kidneys were collected after three hours. Cytokines were analyzed using enzyme-immuno assays and qPCR. In plasma, a substantial increase in interleukin-6 (IL-6), KC (IL-8 analogue), IL-12, monocyte chemoattractant protein (MCP-1), macrophage inflammatory protein MIP-1α, MIP-1β, eotaxin, RANTES and G-CSF (median 90-fold increase) were observed in BD animals compared to sham (all *p* < 0.01). In kidneys, BD substantially induced IL-6, KC, TNF, MCP-1, P-Selectin, and VCAM-1 (all *p* < 0.01). In plasma, C5 and CD14 inhibition, either single or in combination, virtually abolished all cytokines in the BD animals (> 90% for six cytokines and 70–90% for three) (all *p* < 0.01). In kidneys, the effect of inhibition was similar (> 90% for IL-6 and KC and 60–80% for TNF and MCP-1 (all *p* < 0.01). Single and combined inhibition of C5 and CD14 efficiently prevented BD-induced systemic inflammation and reduced local kidney inflammation in a mouse model.

## Introduction

Brain death (BD) induces a potentially harmful systemic inflammation, which may reduce organ quality for transplantation. The inferiority of organs from BD donors compared to living donors is reflected by impaired graft survival and patient outcome and related to the inflammatory response induced by brain injury^1^. More than two decades ago it was shown that BD in rat kidney isografts induced a marked innate immune response including cytokine release and complement activation^2^. Similar results were found in large animals like dogs^3^, pigs^4^, and macaques^5^. In humans, BD and living kidney donors were compared using both systemic blood and organ samples analyzed for a series of inflammatory markers, documenting the substantial brain damage-induced inflammation^6^. Furthermore, samples after reperfusion of human kidneys from BD donors showed a marked increase in cytokines, such as G-CSF, IL-6, IL-9, IL-16 and MCP-1. This is in contrast to kidneys from living and controlled donation after circulatory death donors where a negligible cytokine response was found^7^, consistent with experimental data from mice^8^.

A time-course study in rats showed a massive inflammatory response revealed by the increase in gene expression and proteins (IL-6, MCP-1, KC, E-selectin), as well for the protective genes HO-1 and HSP70, and neutrophil infiltration already 30 min after BD death^9^. This was in contrast to the reactive oxygen species formation which was detectable only in the later observation period of four hours. Several groups have investigated possible early therapeutic interventions to attenuate inflammation, including Selectin inhibitors combined with sirolimus and cyclosporin^10^, carbamylated erythropoietin^11^, sphingosine-1-phosphate 1 receptor agonist^12^, methylprednisolone^13^, NLRP3 inhibition^14^ and angiotensin converting enzyme 2 activation^15^, with significant, though moderate effect.

Innate immunity is the first line of defense, sensing danger, and the main recognition systems are the complement system and the Toll-like receptors (TLRs). Both the complement system and the TLRs have been shown to contribute to the inflammatory response induced by BD. In a mouse study of heart transplantation it was shown that BD exacerbated complement-dependent ischemia/reperfusion injury in the transplanted heart^8^, and in another mice study the lung injury was shown to be complement dependent and primarily induced by the classical and lectin pathway^16^. Blocking of complement factor B reduced renal injury and inflammation in a rat model^17^ and inhibition of TLR4 and TLR2 in rats reduced the BD inflammatory storm, TLR4 inhibition being more efficient that TLR2 inhibition^18^. The crosstalk between complement and TLRs in BD death has been highlighted^19^.

In a human study of kidney transplantation, plasma complement C5a was found to increase in BD donors accompanied by an increased expression of the C5aR in kidney biopsies^20^. Incubation of C5a on the tissue biopsies induced expression of IL-1β, IL-6 and IL-8, supporting a role for the C5a-C5aR axis in the pathogenesis of BD damage to the donor kidney.

Based on our previous studies on dual blockade of complement and TLRs, i.e. the co-receptor CD14 used by both TLR4 and TLR2, to attenuate systemic inflammation^21^, we hypothesized that an upstream combined inhibition of complement component C5 and the CD14 molecule would attenuate the downstream BD-induced inflammatory response.

## Materials and methods

Male WT C57/BL6 mice were bred in the local animal facility at the University Medical Center Groningen and received humane care in compliance with the ‘Principles of Laboratory Animal Care’ and the ‘Guide for the Care and Use of Laboratory Animals’. The experimental protocol was approved by the local animal ethics committee according to the Experiments on Animals Act (CCD approval number AVD1050020171245) and the study is reported according to ARRIVE guidelines. We confirm that all methods and experiments were performed in accordance with the relevant guidelines and regulations.

Mice between 8 and 12 weeks of age, with a weight of 25–28 g were used. The experimental protocol was approved by the local animal ethics committee according to the Experiments on Animals Act.

### Principles of the study

BD was induced with a saline-filled intracranial, extradural balloon. Prior to BD, mice were left untreated (*n* = 8), treated with the C5 inhibitor coversin (*n* = 7), a CD14 inhibitor mAb biG 53 (*n* = 7), or both inhibitors (*n* = 7). Sham mice did not experience BD and were left untreated (*n* = 8). Blood and kidney tissue were collected three hours after BD. Plasma cytokines were analyzed using multiplex technology and kidney mRNA expression examined by qPCR.

### Inhibitors

These have been described in detail in a previous mouse study undergoing cecal ligation and puncture induced sepsis^22^. Briefly, coversin, the recombinant *Ornithodoros moubata* C inhibitor (OmCI), completely prevents C5 cleavage, and blocks complement-mediated C5a and C5b-9 generation completely with the doses used in the present study. Coversin was a kind gift from Akari Therapeutics Plc, London, UK. The anti-mouse CD14 Ab clone biG 53 was produced in CD14 knockout mice and inhibits ligand binding to CD14. The IgG2a isotype was purchased from Biomedtec (Greifswald, Germany) and cleaved into F(ab′)_2_ (Genovis, Lund, Sweden). The F(ab′)_2_ was highly pure as determined by SDS-PAGE and the dose needed for complete CD14 neutralization in vivo was determined^22^. This dose was used in the present study. The coversin-treated group received 100 µg coversin/mouse before BD and the anti-CD14–treated group received 100 µg anti-CD14 F(ab′)_2_/mouse. The combined coversin and anti-CD14 group received the same doses as the individual groups. The positive control (BD) and the Sham group received Dulbecco’s PBS (DPBS).

### Brain death model

BD was performed as described previously by Atkinson et al.^8,23^. Briefly, mice were anesthetized using isoflurane, intubated and ventilated. The carotid artery was cannulated to measure the mean arterial pressure (MAP) and the jugular vein was cannulated to infuse fluids and medications. A hole was drilled in the frontolateral part of the skull, and a balloon catheter was inserted to an intracranial, extradural position. Brain death was induced by increasing the intracranial pressure through inflation of the balloon. BD was confirmed by absence of corneal reflexes and a positive apnea test (no breathing for 1 min). BD was maintained for 3 h, in which the MAP was kept above 60 mmHg. 50 µl of 0.9% saline combined with 50 µg/ml lepirudin was administered every 15 min or upon drop of MAP < 60mmHg to a maximum of 1.2 ml. In sham-operated mice, a hole was drilled in the skull, without insertion of a balloon. Blood, urine and kidneys were collected after 3 h of brain death.

### Laboratory analyses

*Plasma cytokines.* Twenty-three cytokines, including chemokines and growth factors, were measured by a multiplex assay from Bio-Rad (Hercules, CA). The assay included the following analytes: IL-1α, IL-1β, IL-2, IL-3, IL-4, IL-5, IL-6, IL-9, IL-10, IL-12 p40, IL-12 p70, IL-13, IL-17, eotaxin (CCL11), G-CSF, GM-CSF, IFN-γ, keratinocyte chemoattractant (CXCL1, KC, IL-8 analogue), MCP-1 (CCL2), macrophage inflammatory protein-1α (MIP-1α; CCL3), MIP-1β (CCL4), RANTES (CCL5), and TNF. The analysis was preformed according to the manufacturer’s instructions.

*Plasma creatinine.* Creatinine was measured by Creatinine Competitive LISA kit, documented to react with mouse creatinine as well as a number of other species, including humans (ThermoFisher, Bender Medsystems GmbH, Vienna, Austria). The procedure was performed according to the manufacturer’s instructions. All values were on the linear part of the standard curve given in µg/mL.

*Kidney tissue.* RT-qPCR was performed to detect the level of pro-inflammatory gene expression (TNF, IL-6, KC (IL-8) and MCP-1) and the adhesion molecules VCAM-1 and P-selectin in donor kidneys. Total RNA was extracted from frozen kidneys using TRIzol (Invitrogen Life Technologies, Breda, the Netherlands), according to manufacturer’s instructions. RNA integrity was confirmed by gel electrophoresis and DNAse I (Invitrogen) was used to remove genomic DNA. Extracted RNA was reverse transcribed using M-MLV Reverse Transcriptase (Invitrogen Life Technologies) according to manufacturer’s instructions. The Taqman Applied Biosystems 7900HT RT-qPCR system (Applied Biosystems, Carlsbad, CA) was used to amplify and detect RT-qPCR products, by measuring SYBR green (Applied Biosystems) emission using specific primers (Table 1). Thermal cycling was initiated with a hot start on.

**Table 1:** Primer sequences for qRT-PCR in kidney tissue.

Gene	Forward sequence (5′ → 3′)	Reverse sequence (5′ → 3′)
TNF	AATAACGCTGATTTGGTGACCAG	GATTACAGTCACGGCTCCCGT
IL-6	ACATAAAATAGTCCTTCCTACCCCAATT	TTAGCCACTCCTTCTGTGACTCC
KC (IL-8)	GTGTCTAGTTGGTAGGGCATAATGC	TGTCCCGAGCGAGACGAG
MCP-1	TTCAACACTTTCAATGTATGAGAGATGA	AACAATACCTTGGAATCTCAAACACA
VCAM-1	CCCGAACTCCTTGCACTCTACT	CCCGATGGCAGGTATTACCA
P-sel	CCTCACAGCCACCTAGGAACA	GTTGGGTCATATGCAGCGTTAG
b-act	ACACCCTTTCTTTGACAAAACCTAA	GCCATGCCAATGTTGTCTCTTAT

50 ⁰C and increased to 95 ⁰C for denaturation. Thereafter, the annealing step and DNA synthesis were achieved after 40 repeated cycles at 60 ⁰C. Generation of single, specific amplicons were confirmed by melt curve analyses. CT-values were corrected for house-keeping gene β-actin.

### Statistics

The five animal groups were compared using One-way ANOVA with post-hoc Tukey correction for multiple testing. One extreme outlier for TNF was excluded (> 3 times higher than the SD), regarded to be a technical error. Data are presented as mean with individual animals (dots) and with 95% Confidence Interval indicated in Results.

## Results

### Plasma cytokines measured my multiplex

Of the 23 cytokines measured in plasma, the following nine increased significantly (all *p* < 0.01) in the BD group as compared to sham and were included in the further analyses: interleukin-6 (IL-6), KC (IL-8 analogue), IL-12, monocyte chemoattractant protein (MCP-1), macrophage inflammatory protein (MIP)-1α, MIP-1β, eotaxin, RANTES and G-CSF (all *p* < 0.01).

**Fig. 1:**
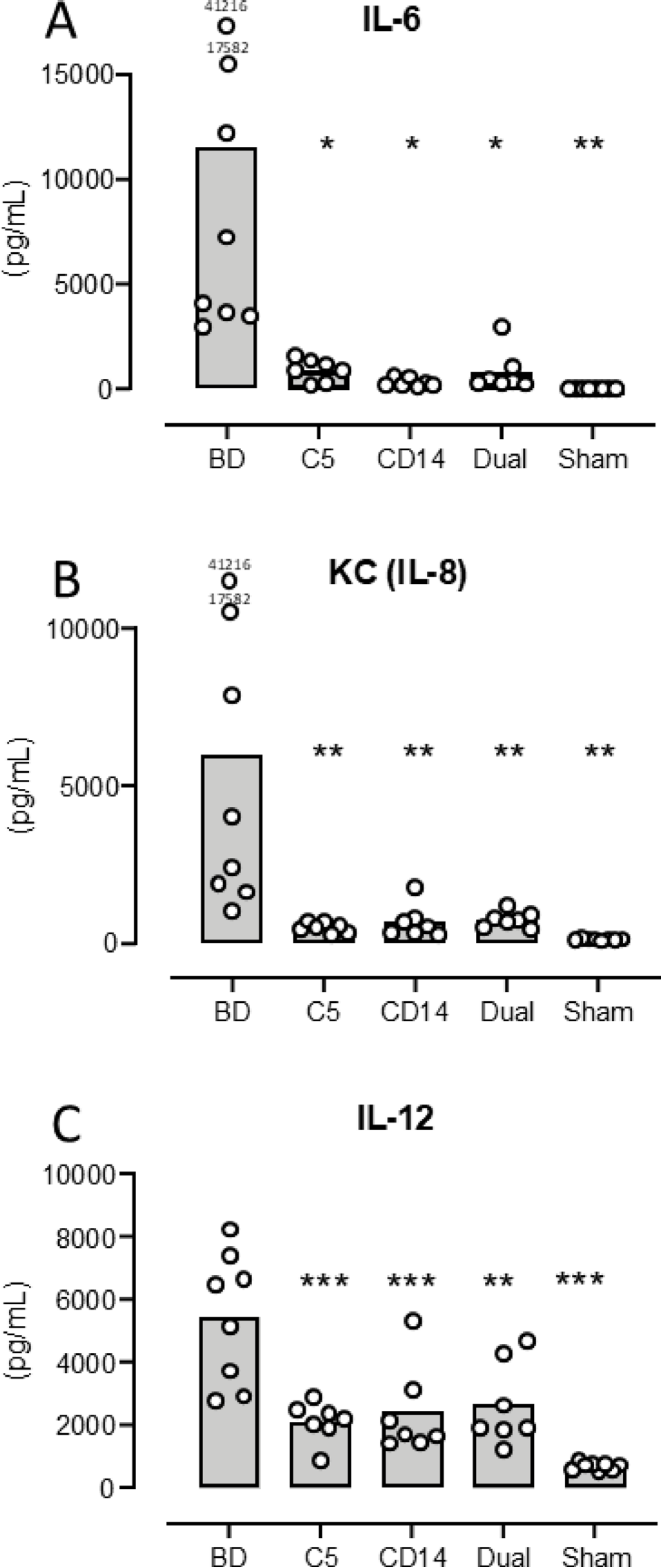
Plasma concentration of cytokines in mice three hours after brain death - I. Plasma IL-6 (**A**), KC (IL-8) (**B**) and IL-12 (**C**) concentrations as measured by multiplex technology are shown for the five animal groups: brain death (BD), the C5 inhibitor coversin (C5), the inhibitory anti-CD14 mAb biG 53 (CD14), the combination of the two inhibitors (Dual) and the Sham. Significances are compared with BD. Data are presented as mean and dots for the individual animals. * *p* < 0.05, ** *p* < 0.01, *** *p* < 0.001.

***IL-6*** increased from mean 5.8 pg/mL (95% CI: 4.3–7.4) in sham to 11,565 pg/mL (656-22473) (*p* = 0.006) in the BD group (Fig. 1A). The IL-6 increase was significantly lower in the treated groups compared to the BD group: C5 inhibition group 924 pg/mL (443–1407) (*p* = 0.016), CD-14 inhibition group 327 pg/mL (141–513) (*p* = 0.010) and the dual treatment group 825 pg/mL (-93-1744) (*p* = 0.015) (Fig. 1A).

***KC*** increased from mean 117 pg/mL (98–136) in sham to 6009 pg/mL (1148–10869) (*p* = 0.001) in the BD group (Fig. 1B). The KC increase was significantly lower in the treated groups compared to the BD group: Coversin group 502 pg/mL (356–658) (*p* = 0.004), anti-CD14 group 666 pg/mL (185–1147) (*p* = 0.006) and the dual treatment group 744 pg/mL (511–977) (*p* = 0.006) (Fig. 1B).

***IL-12*** increased from mean 710 pg/mL (606–815) in sham to 5430 pg/mL (3686–7174) (*p* < 0.0001) in the BD group (Fig. 1C). The IL-12 increase was significantly lower in the treated groups compared to the BD group: Coversin group 2126 pg/mL (1526–2706) (*p* = 0.0003), anti-CD14 group 2422 pg/mL (1119–3725) (*p* = 0.001) and the dual treatment group 2658 pg/mL (1430–3885) (*p* = 0.003) (Fig. 1C).

**Fig. 2:**
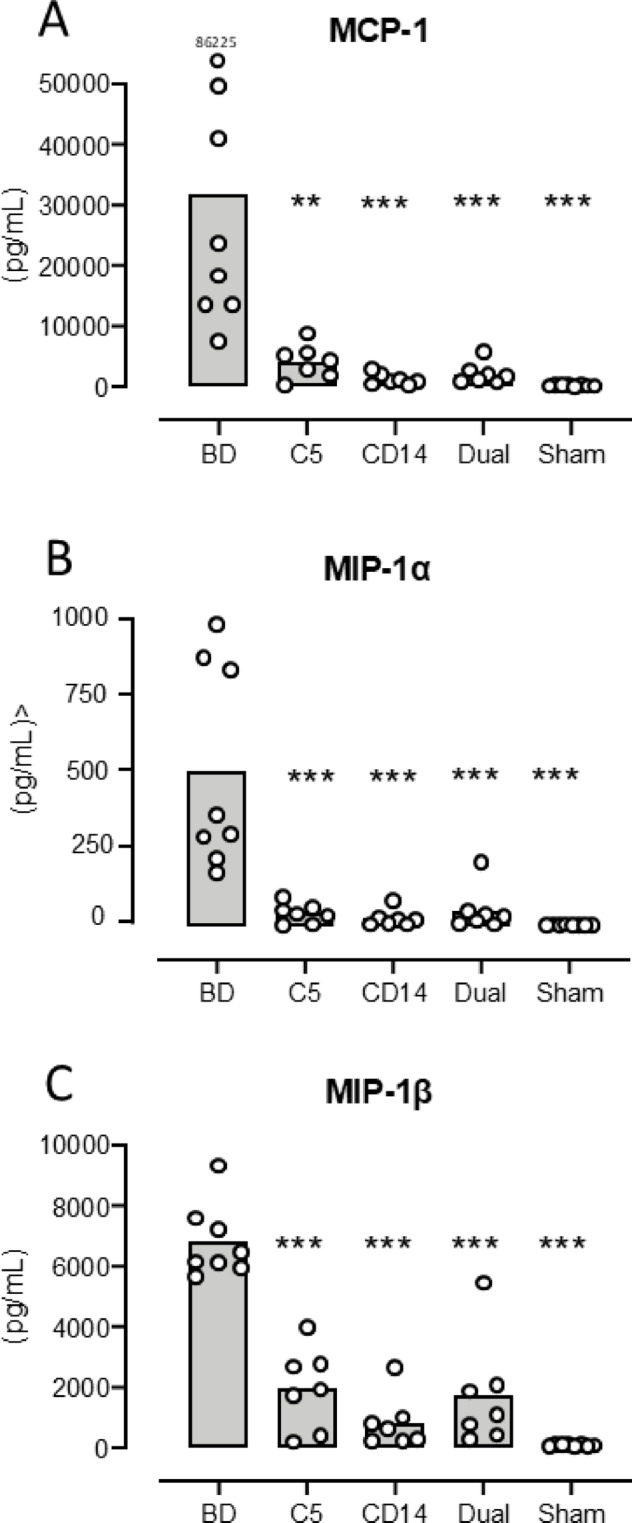
Plasma concentration of cytokines in mice three hours after brain death - II. Plasma MCP-1 (**A**), MIP-1α (**B**) and MIP-1β (**C**) concentrations as measured by multiplex technology are shown for the five animal groups: brain death (BD), the C5 inhibitor coversin (C5), the inhibitory anti-CD14 mAb biG 53 (CD14), the combination of the two inhibitors (Dual) and the Sham. Significances are compared with BD. Data are presented as mean and dots for the individual animals. ** *p* < 0.01, *** *p* < 0.001.

***MCP-1*** increased from mean 269 pg/mL (218–321) in sham to 31,718 pg/mL (9696–53740) (*p* = 0.0001) in the BD group (Fig. 2A). The MCP-1 increase was significantly lower in the treated groups compared to the BD group: Coversin group 4227 pg/mL (1663–6792) (*p* = 0.001), anti-CD14 group 1284 pg/mL (433–2135) (*p* = 0.0004) and the dual treatment group 2215 pg/mL (555–3875) (*p* = 0.0006) (Fig. 2A).

***MIP-1α*** increased from mean 6.8 pg/mL (5.7-8.0) in sham to 540 pg/mL (244–836) (*p* < 0.0001) in the BD group (Fig. 2B). The MIP-1α increase was significantly lower in the treated groups compared to the BD group: Coversin group 47 pg/mL (15–79) (*p* < 0.0001), anti-CD14 group 28 pg/mL (2.3–54) (*p* < 0.0001) and the dual treatment group 57 pg/mL (-12-126) (*p* < 0.0001) (Fig. 2B).

***MIP-1β*** increased from mean 98 pg/mL (75–121) in sham to 6808 pg/mL (5799–7817) (*p* < 0.0001) in the BD group (Fig. 2C). The MIP-1β increase was significantly lower in the treated groups compared to the BD group: Coversin group 1967 pg/mL (725–3210) (*p* < 0.0001), anti-CD14 group 839 pg/mL (53-1625) (*p* < 0.0001) and the dual treatment group 1721 pg/mL (72-3369) (*p* < 0.0001) (Fig. 2C).

**Fig. 3:**
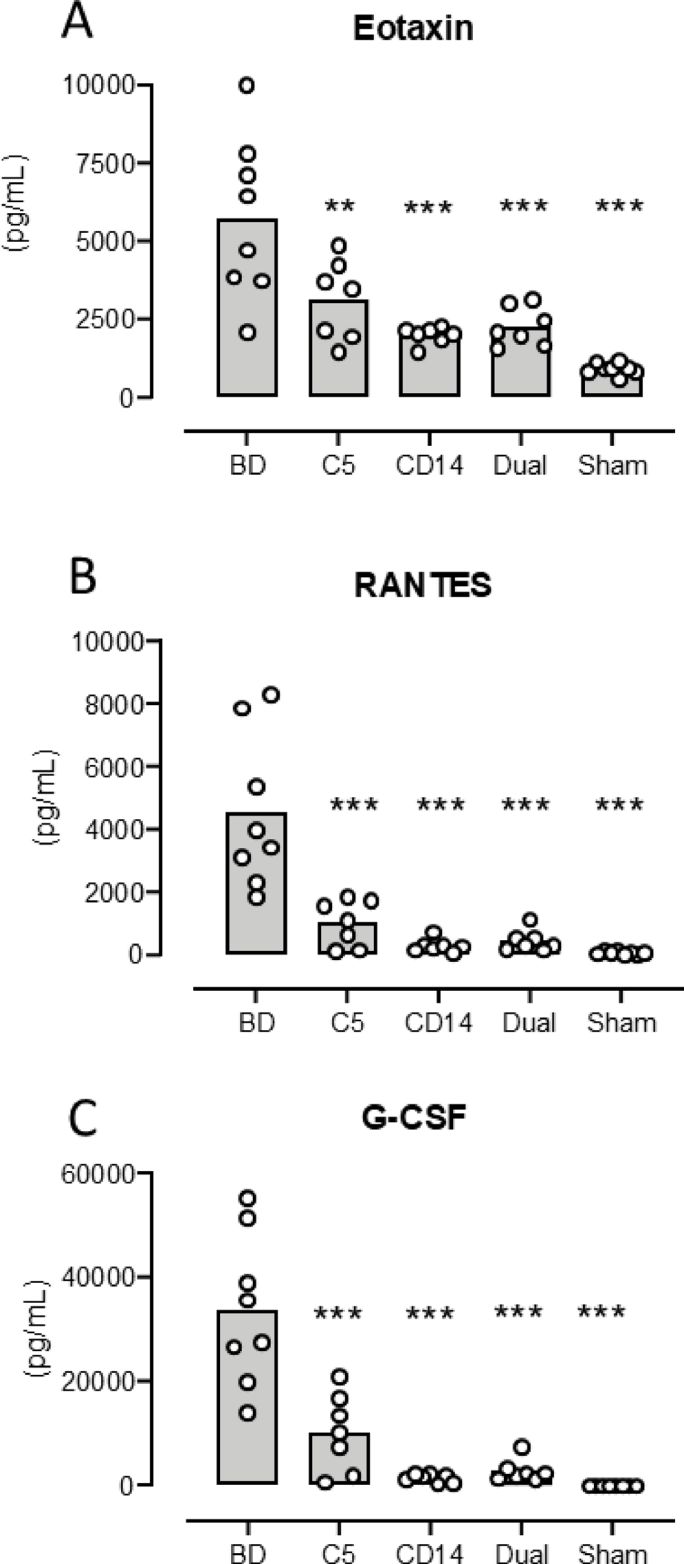
Plasma concentration of cytokines in mice three hours after brain death - III. Plasma eotaxin (**A**), RANTES (**B**) and G-CSF (**C**) concentrations as measured by multiplex technology are shown for the five animal groups: brain death (BD), the C5 inhibitor coversin (C5), the inhibitory anti-CD14 mAb biG 53 (CD14), the combination of the two inhibitors (Dual) and the Sham. Significances are compared with BD. Data are presented as mean and dots for the individual animals. ** *p* < 0.01, *** *p* < 0.001.

***Eotaxin*** increased from mean 908 pg/mL (753–1063) in sham to 5733 pg/mL (3533–7933) (*p* < 0.0001) in the BD group (Fig. 3A). The eotaxin increase was significantly lower in the treated groups compared to the BD group: Coversin group 3105 pg/mL (1921–4289) (*p* = 0.007), anti-CD14 group 1933 pg/mL (1741–2246) (*p* < 0.0001) and the dual treatment group 2256 pg/mL (1692–2821) (*p* = 0.0003) (Fig. 3A).

***RANTES*** increased from mean 50 pg/mL (17–84) in sham to 4515 pg/mL (2470–6561) (*p* < 0.0001) in the BD group (Fig. 3B). The RANTES increase was significantly lower in the treated groups compared to the BD group: Coversin group 1004 pg/mL (331–1677) (*p* < 0.0001), anti-CD14 group 267 pg/mL (75–460) (*p* < 0.0001) and the dual treatment group 420 pg/mL (107–733) (*p* = 0.001) (Fig. 3B).

***G-CSF*** increased from mean 25 pg/mL (18–32) in sham to 33,598 pg/mL (21488–45708) (*p* < 0.0001) in the BD group (Fig. 3C). The G-CSF increase was significantly lower in the treated groups compared to the BD group: Coversin group 10,224 pg/mL (328-17166) (*p* < 0.0001), anti-CD14 group 1412 pg/mL (671–2152) (*p* < 0.0001) and the dual treatment group 2855 pg/mL (867–4843) (*p* < 0.0001) (Fig. 3C).

All three treatment groups showed statistic significant inhibition of all the nine cytokines and there was no significant difference between the three groups for any of the cytokines. The magnitude of the inhibition was calculated in percentage reduction with treatment as compared with the BD group. IL-6, KC, MCP-1, MIP-1α, RANTES and G-CSF were inhibited by > 90%, IL-12, MIP-1β and eotaxin in the range of 70–90%.

### Kidney tissue cytokines measured by qPCR

In kidneys, BD significantly induced TNF, IL-6, KC, MCP-1, VCAM-1 and P-selectin (all *p* < 0.01).

**Fig. 4:**
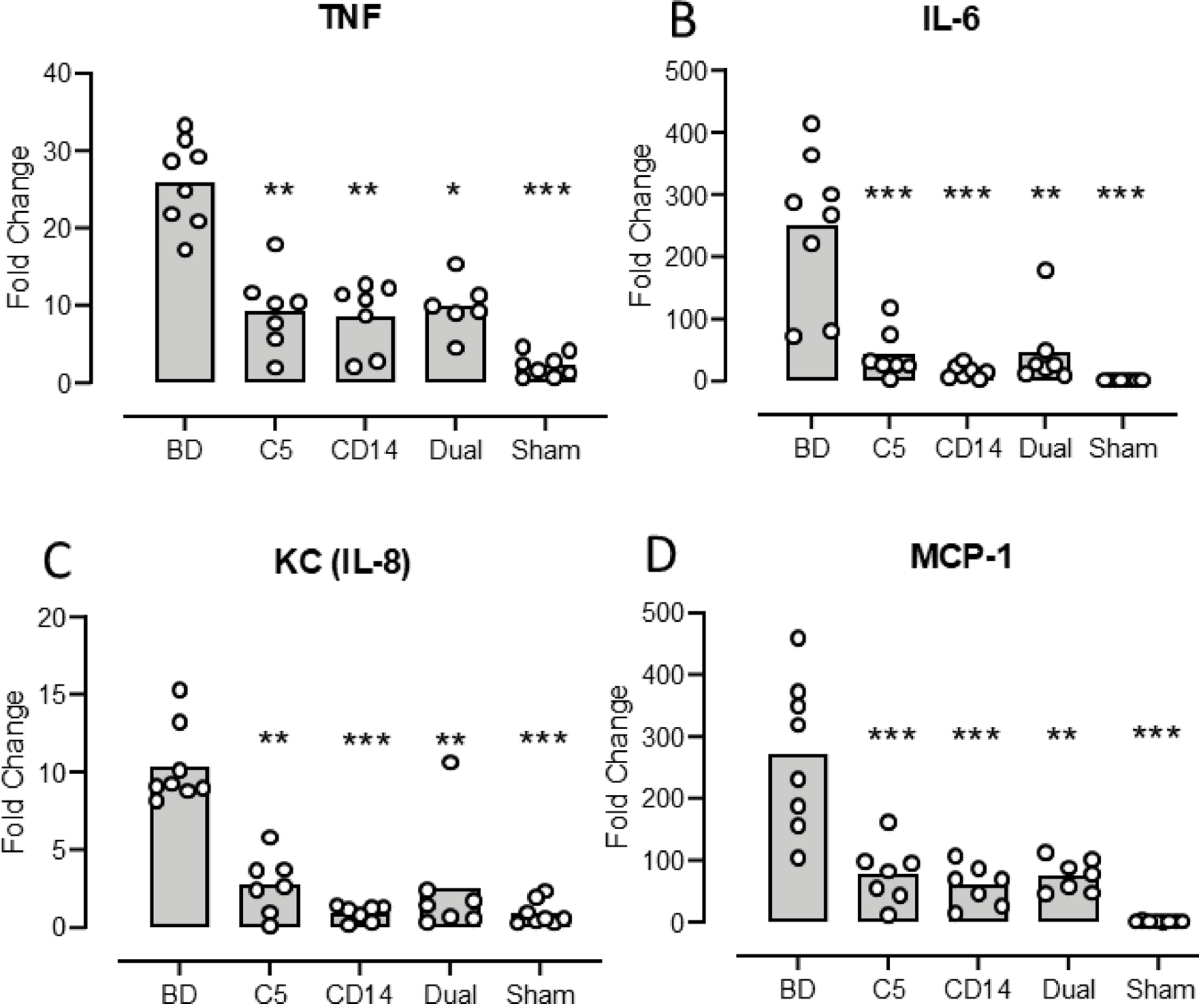
Kidney expression of cytokines in mice three hours after brain death. Kidney TNF (**A**), IL-6 (**B**), KC (IL-8) (**C**) and MCP-1 (**D**) as measured by qPCR are shown with fold change for the five animal groups: brain death (BD), the C5 inhibitor coversin (C5), the inhibitory anti-CD14 mAb biG 53 (CD14), the combination of the two inhibitors (Dual) and the Sham. Significances are compared with BD. Data are presented as mean and dots for the individual animals. * *p* < 0.05, ** *p* < 0.01, *** *p* < 0.001.

***TNF*** increased from mean fold change (FC) 2.3 (1.0-3.5) in sham to 26 (21–31) (*p* < 0.0001) in the BD group (Fig. 4A). The TNF increase was significantly lower in the treated groups compared to the BD group: Coversin group FC 9.4 (4.7–14) (*p* = 0.009), anti-CD14 group FC 8.7 (4.5–12) (*p* = 0.004) and dual treatment group FC 9.9 (6.2–13) (*p* = 0.036) (Fig. 4A).

***IL-6*** increased from mean FC 0.73 (0.57–0.88) in the sham to FC 251 (43–123) (*p* < 0.0001) in the BD group (Fig. 4B). The IL-6 increase was significantly lower in the treated groups compared to the BD group: Coversin group FC 43 (6–79) (*p* = 0.0005), anti-CD14 group FC 15 (4.9–24) (*p* < 0.0001) and the dual treatment group FC 45 (-10-100) (*p* = 0.0007) (Fig. 4B).

***KC*** increased from mean FC 0.9 (0.3–1.6) in the sham to FC 10 (8.3–13) (*p* < 0.0001) in the BD group (Fig. 4C). The KC increase was significantly lower in the treated groups compared to the BD group: Coversin group FC 2.8 (1.0-4.5) (*p* = 0.009), anti-CD14 group FC 0.9 (0.5–1.4) (*p* < 0.0001) and the dual treatment group FC 2.5 (-0.8-5.9 (*p* = 0.002) (Fig. 4C).

***MCP-1*** increased from mean FC 1.4 (0.9–1.9) in the sham to FC 272 (170–374) (*p* < 0.0001) in the BD group (Fig. 4D). The MCP-1 increase was significantly lower in the treated groups compared to the BD group: Coversin group FC 77 (33–121) (*p* = 0.0006), anti-CD14 group FC 59 (29–90) (*p* < 0.0001) and the dual treatment group FC 76 (51–100) (*p* = 0.003) (Fig. 4D).

All three treatment groups showed statistic significant inhibition of all the four cytokines and there was no significant difference between the three groups for any of the cytokines. The magnitude of the inhibition was calculated in percentage reduction with treatment as compared with the BD group. IL-6 and KC were inhibited by > 90% and TNF and MCP-1 in the range of 60–80%.

### Kidney tissue adhesion molecules measured by qPCR

**Fig. 5:**
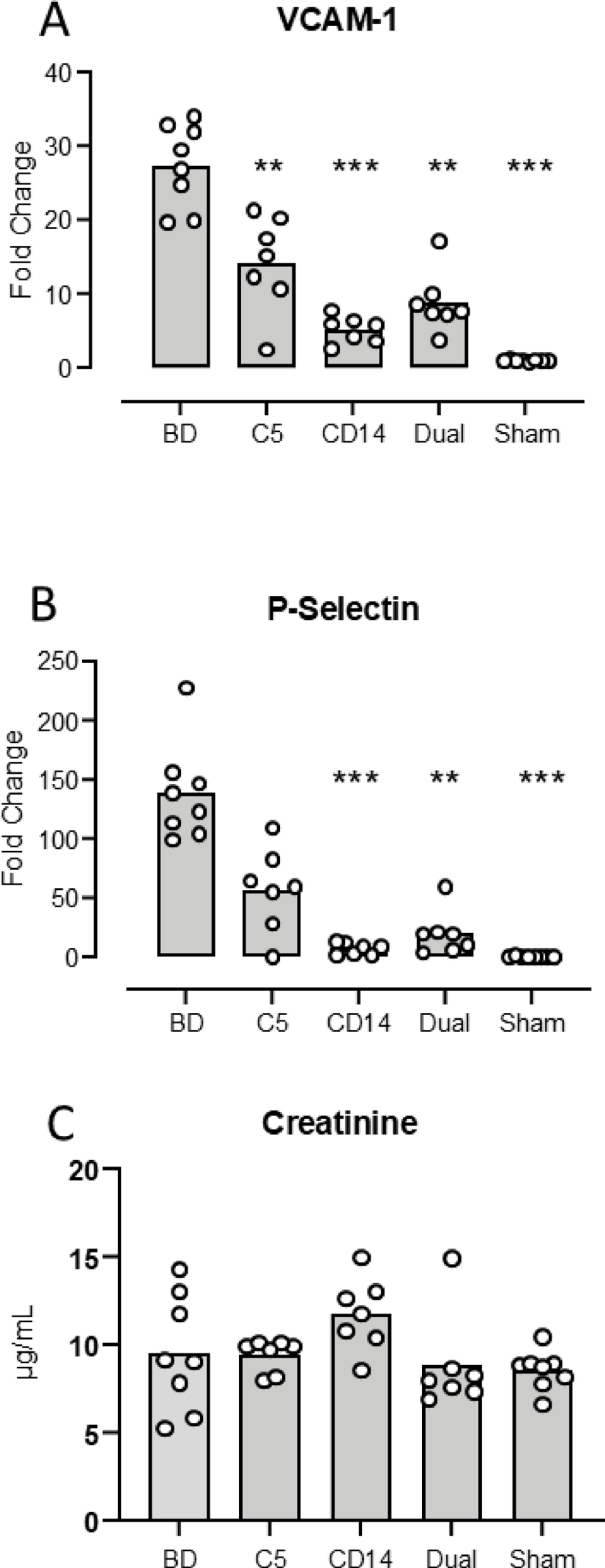
Kidney expression of adhesion molecules in mice three hours after brain death. Kidney VCAM-1 (**A**) and P-Selectin (**B**) as measured by qPCR are shown with fold change for the five animal groups: brain death (BD), the C5 inhibitor coversin (C5), the inhibitory anti-CD14 mAb biG 53 (CD14), the combination of the two inhibitors (Dual) and the Sham. Plasma levels of creatinine is shown in panel C. Significances are compared with BD. Data are presented as mean and dots for the individual animals. ** *p* < 0.01, *** *p* < 0.001.

***VCAM-1*** increased from mean FC 1.1 (1.1–1.2) in the sham to FC 27 (23–32) (*p* < 0.0001) in the BD group (Fig. 5A). The VCAM-1 increase was significantly lower in the treated groups compared to the BD group: Coversin group FC 14 (8.3–20) (*p* = 0.008), anti-CD14 group FC 5.3 (3.6–6.9) (*p* < 0.0001) and the dual treatment group FC 8.9 (5.1–13) (*p* < 0.0001) (Fig. 5A).

***P-selectin*** increased from mean FC 0.6 (0.1–1.1) in the sham to FC 139 (104–173) (*p* < 0.0001) in the BD group (Fig. 5B). The P-selectin increase was significantly lower in the anti-CD14 treated group, FC 7.4 (2.8–12) (*p* < 0.0001), and the dual treatment group, FC 20 (3.0–37) (*p* = 0.0009), compared to the BD group, whereas for the coversin group only a trend was observed, FC 57 (25–89) (*p* = 0.06) (Fig. 5B).

In contrast to the systemic and local cytokines described above (Figs. 1, 2, 3 and 4), the adhesion molecules revealed a difference between the treatment groups. Both VCAM-1 and P-selectin differed significantly between the single treatment groups. CD14 inhibition was significantly more efficient as compared to coversin: *p* = 0.004 for ICAM-1 and *p* = 0.014 for P-selectin. No significant differences were found between CD14 inhibition and combined C5 and CD14 inhibition. The magnitude of the inhibition was calculated in percentage reduction with treatment as compared with the BD group. CD14 inhibition reduced VCAM-1 and P-selectin expression by 83% and 95%, respectively. Coversin reduced VCAM-1 and P-Selectin expression by 48% and 59%, respectively. Dual inhibition reduced VCAM-1 and P-Selectin expression by 67% and 86%, respectively.

***Plasma creatinine*** did not increase in the BD group compared to sham. The values in all 5 group were within the normal range of plasma creatinine (Fig. 5C).

## Discussion

In the present study, we show that inhibition of complement component C5 and the TLR co-receptor CD14 in mice undergoing BD substantially reduced systemic cytokine release and kidney specific cytokine and adhesion molecule expression for most by > 90%. Complement and TLRs represent two main branches of innate immunity activated through their pattern recognition receptors (PRRs) to induce a cytokine storm. To the best of our knowledge this is the first study with a therapeutic regimen inhibiting both these systems simultaneously, leading to a substantial suppression of the innate immune response induced by BD.

We have been working on a hypothesis^24^ that two of the main recognition branches of innate immunity to sense danger, the complement system and the TLRs, are the main inducers of the detrimental inflammatory reaction to both infectious pathogen associated molecular patterns (PAMPs) and to none-infectious (sterile) tissue damage associated molecular patterns (DAMPs). Complement and TLRs respond to DAMPs and PAMPs by their pattern recognition receptors (PRRs) and induce a down-stream pan-inflammatory response including release of e.g. cytokines, reactive oxygen species, arachidonic acid metabolites, and expression of leukocyte, platelet and endothelial cell surface molecules. Thus, we searched for an upstream “bottle-neck” approach to block the systemic and detrimental overactivation of innate immunity when this activation, more than the underlying cause, threatens the host, like in sepsis and trauma^21^. In the present study we forwarded this hypothesis of the dual blockade to be relevant also for the cytokine storm occurring in the course of BD.

To define central “bottle-neck” molecules to block the upstream complement and TLR systems, we investigated the role of the central complement components C3 and C5, and the important co-receptor CD14 in the TLR system, the latter documented to being essential for TLR4 and TLR2 in humans and also for three other TLRs in mice^25^. We have developed an ex vivo human whole blood model^26^ to investigate the role of complement and CD14 in inducing the downstream inflammatory response. In a number of studies, we have documented that this dual blockade is highly potent in inhibiting the inflammatory response, including the cytokine storm, to PAMPs, i.e. Gram-negative (*Eschericia coli*,* Neisseria meningitidis)* and Gram-positive bacteria (*Staphylococcus aureaus)*^27–32^. These studies indicated that inhibition of C3 or C5 were similar with respect to the inflammatory response induced by complement, consistent with previous studies showing C5a to be the most important inflammatory inductor in mice sepsis^33^ and in human Covid infection^34^.

Thus, we decided for a complement C5 and a CD14 inhibitor in the present study of BD, representing a sterile DAMP-induced inflammatory response. C5 inhibition blocks release of the anaphylatoxin C5a, the most potent inflammatory mediator of complement activation, including release of cytokines. CD14 inhibition blocks both TLR4 and TLR2 and serves as an upstream TLR system “bottle neck” inhibitor. The complement and the TLR systems, activates the inflammatory response after brain death through their recognition of innumerable of DAMPs (endogenous “alarmins”) released by leukocytes and number of other cells in blood and various organs. Some of the DAMPs act as ligands for the TLRs including HMBG-1, heat shock proteins, fibronectin, hyaluronic acid, heparan sulfate, S100, histones and nucleic acids. The most important observation in the present study was the substantial inhibition of the cytokine release, reaching > 90% reduction in most of the cytokines systemically and in the kidney, while all cytokines were reduced by > 60% by inhibition of C5 or CD14. In addition, no significant differences between intervention groups and sham animals were found, implying that dual inhibition may reveal systemic and tissue specific cytokine expression comparable to living kidney donation.

We observed that the single inhibition of C5 and CD14 was virtually identical with respect to inhibition of the cytokines, and the combined inhibition did not add any significant effect. This is in contrast to what we have observed in ex vivo studies in a human whole blood model incubated with bacteria, where a differential effect has been seen for C5 and CD14 inhibition^28,30,35^. In these former studies, IL-6 was very efficiently reduced by inhibiting CD14 and IL-8 by inhibiting complement, but importantly dual blockade was much more efficient than singe inhibition and reduced the response by > 90. The present data are more in accordance with results from a previous mice sepsis study^22^. Here, we observed substantial effect on the cytokine response with either C5 or CD14 inhibition. However, there was still a significant additional effect of the dual blockade. There may be several explanations for the differential effect of C5, CD14 and combined inhibition. Species differences in the innate immune response between mice, pigs and humans may be one explanation, e.g. impact of differences in the redundancy between the branches of the inflammatory network. The differences between the two mice studies may be explained by the two the models. The systemic inflammatory response induced by PAMPs in sepsis and DAMPs in brain death, with different cytokine patterns, may respond differently to the inhibitory regimens. Thus, in future studies, both in animals and in humans, inhibition of C5 and CD14, and their combination, should be considered.

In contrast to the cytokines, the adhesion molecules in the kidney responded differently to C5 and CD14 inhibition. Inhibition of CD14 was more efficient in attenuating VCAM-1 and P-selectin expression. CD14 inhibition substantially and significantly reduced both molecules, whereas C5 inhibition resulted in a lesser reduction which was not significant for P-selectin, though still approximately 50%. This underscores a possible different effect of inhibition of complement and TLRs on cytokines versus adhesion molecules. This needs further investigation and emphasizes at the same time to take both systems in consideration. Thus, we argue that future studies should evaluate the effect of dual inhibition of C5 and CD14.

We decided to use the same dose of inhibitors as described in a mouse model of sepsis^22^. The dose was titrated for this sepsis model. Sepsis is one of the most powerful inflammatory models for studying innate immunity, including complement and TLRs, with both PAMPs and DAMPs contributing. BD is regarded to be a less powerful inflammatory response, limited to DAMPS only. The fact that we observed a virtually complete inhibition of cytokines by on average of 90% of the cytokines, implies that the dose was sufficient.

Plasma creatinine did not change in any of the groups and was indeed similar in the BD and the sham groups, indicating that during the observation period of three hours the kidney function as measured by creatinine was stable and not affected by brain death. This is not surprising, due to the short observation period but does not exclude detrimental pathophysiological processes going on, as reflected by the increase in cytokines in the kidney, most likely leading to an inflammatory reaction that later may progress to a reduction in kidney function.

### Limitations

This study would have profited from examining different populations of leukocytes both with respect to activation and analysis of surface markers, as well as the mechanisms of the cytokine release. Furthermore, serial samples for investigation of a number of analytes to follow the timeline after BD would have been informative. Unfortunately, the amount of blood available, the logistics, the risk of blood sampling affecting the animal and sensitive inflammatory read-outs precluded us for including more readouts and biomarkers than those presented in this article. By choosing one single main endpoint at three hours, we succeeded in mice surviving this period, and we got enough blood and high-quality tissue samples for the most important analyses. Indeed, a single endpoint observation is a limitation of the study and explains why organ damage could not be revealed, i.e. kidney function. However, three hours observation period were optimal for the induction of the inflammatory response, and this response may lead to subsequent organ damage. Furthermore, we did not collect baseline blood samples as we did not want to introduce possible inflammatory stimuli. Thus, we regard the sham group who did not underwent brain death as control.

In conclusion, the innate immune system represented by complement and TLRs is crucial for inducing inflammatory reactions in the course of BD. Inhibition of C5 and CD14 efficiently prevented BD induced inflammation both systemically and locally in the kidney. Complement- and CD14 inhibitors are available for clinical use and clinical studies should be performed with deceased brain death donors to enhance donor organ quality.

## Data Availability

The datasets generated during and/or analysed during the current study are available from the corresponding author on reasonable request.
